# Epidemiology and Outcomes of Infected Non-Unions: An Observational Study at an Infectious Disease Referral Centre

**DOI:** 10.3390/antibiotics13121180

**Published:** 2024-12-05

**Authors:** Sara Tedeschi, Nicolò Rossi, Eleonora Zamparini, Simone Ambretti, Massimiliano Mosca, Cesare Faldini, Stefano Zaffagnini, Alessandra Maso, Andrea Sambri, Massimiliano De Paolis, Pierluigi Viale

**Affiliations:** 1 Department of Medical and Surgical Sciences, Alma Mater Studiorum University of Bologna, 40126 Bologna, Italy; sara.tedeschi@unibo.it (S.T.); nicolo.rossi@unibo.it (N.R.); simone.ambretti@aosp.bo.it (S.A.); pierluigi.viale@unibo.it (P.V.); 2 Infectious Diseases Unit, Department for Integrated Infectious Risk Management, IRCCS Azienda Ospedaliero-Universitaria di Bologna, 40138 Bologna, Italy; eleonora.zamparini@aosp.bo.it; 3 Microbiology Unit, IRCCS Azienda Ospedaliero-Universitaria di Bologna, 40138 Bologna, Italy; 4 UO Ortopedia Bentivoglio, IRCCS Istituto Ortopedico Rizzoli, 40136 Bologna, Italy; massimiliano.mosca@ior.it; 5 I Orthopaedics and Traumatology Clinic, IRCCS Istituto Ortopedico Rizzoli, 40136 Bologna, Italy; cesare.faldini@unibo.it; 6 Department of Biomedical and Neuromotor Science-DIBINEM, Alma Mater Studiorum University of Bologna, 40126 Bologna, Italy; stefano.zaffagnini@unibo.it; 7 II Clinic of Orthopaedics and Traumatology, IRCCS Istituto Ortopedico Rizzoli, 40136 Bologna, Italy; 8 Laboratory of Microbiology and GMP Quality Control, IRCCS Istituto Ortopedico Rizzoli, 40136 Bologna, Italy; alessandra.maso@ior.it; 9 Orthopaedics and Traumatology Unit, IRCCS Azienda Ospedaliero-Universitaria di Bologna, 40138 Bologna, Italy; massimiliano.depaolis@aosp.bo.it

**Keywords:** infected, non-union, pseudoarthrosis

## Abstract

**Objectives**: The main aim of this study was to describe the epidemiology of infected non-unions (INUs) managed at an Infectious Disease (ID) referral centre and to investigate the factors associated with treatment failure. **Methods**: This was an observational retrospective study on adult patients with INUs managed between 2012 and 2018 at the ID Unit of the IRCCS Azienda Ospedaliero-Universitaria di Bologna, an Italian ID referral centre for bone and joint infections. Patients were observed for at least 24 months. Those who achieved clinical success were compared with those who experienced clinical failure; to identify factors associated with treatment failure, we performed a univariate and multivariate logistic regression analysis. **Results**: Overall, 78 patients were included. A total of 57/78 (73%) were males; their median age was 43 (IQR 34–56) years; their median Charlson index was 0 (IQR 0–2); 32/78 (41%) reported a history of an open fracture; the non-union most frequently involved the leg. Polymicrobial infection was found in 23/78 cases (29%); the most common microorganisms were coagulase-negative staphylococci (n = 47) and *Staphylococcus aureus* (n = 35). At 24-month follow-up from index surgery, 16/78 patients had experienced clinical failure: 13 (16.6%) presented with persistence of local signs of infection and 3 (3.8%) had undergone amputation. Logistic regression analysis of risk factors for clinical failure identified body mass index (BMI) (aOR 1.15; 95% CI 1.03–1.28, *p* = 0.01) and MRSA infection (aOR 5.35; 95% CI 1.06–26.92, *p* = 0.04) as factors associated with clinical failure. **Conclusions**: Given that a standardized management of antibiotic therapy is initiated by an expert ID consultant team, BMI and MRSA infection are associated with worse outcomes among patients with INUs.

## 1. Introduction

Infected non-unions (INUs) represent one of the most challenging complications in trauma surgery. They usually require multiple surgical procedures and prolonged antibiotic treatment and may result in long-term functional impairment [1]. The overall treatment success rate is 70–90% in high-income countries [2], but patients often experience a substantial reduction in quality of life (functional loss, amputation, chronic pain, depression).

The risk of infection after fracture fixation depends on the baseline characteristics of the patient (e.g., presence of diabetes mellitus, smoking) and the severity of the injury (type of fracture, polytrauma, degree of soft tissue and vascular damage). This risk is around 1% for closed fractures and Gustilo–Anderson grade 1 open fractures, but it is almost 10–15% in Gustilo–Anderson 3B/3C fractures [3,4].

Even with a treatment success rate of 70–90% in high-income countries, patients are often left with a substantial reduction in their quality of life due to permanent functional loss or amputation of the affected limb. Amputation can be inevitable in 3–17% of patients with fracture-related infections, severe comorbidities, or associated bone defects. Aside from chronic pain, physical impairment, and absence from work and home activities, fracture-related infections are associated with a major psychological burden, including depression.

The successful treatment of INUs is based on a combination of surgery (debridement plus implant removal/exchange) and antibiotic therapy. Despite it being recognized that both surgical and medical treatment are needed for the management of INUs, formal guidelines on the medical and surgical treatment of INUs are still lacking and, in clinical practice, management strategies are often extrapolated from those of other implant-related infections, in particular prosthetic joint infections.

The available literature describes union rates after the treatment of an INU ranging from 66 to 100%, with a recurrence of infection in 0–60% of cases [5].

However, the interpretation of previous research in the field has been hampered by the lack of a clear definition of INUs, which was only reached in 2018, and by the absence of management guidelines [5,6,7].

Despite advancements in treating fracture-related infections, there are limited data on the epidemiology and outcomes of infected non-unions managed at referral centres. This study aims to address this gap by providing detailed insights into the risk factors and outcomes of INU management.

## 2. Results

**Figure 1:**
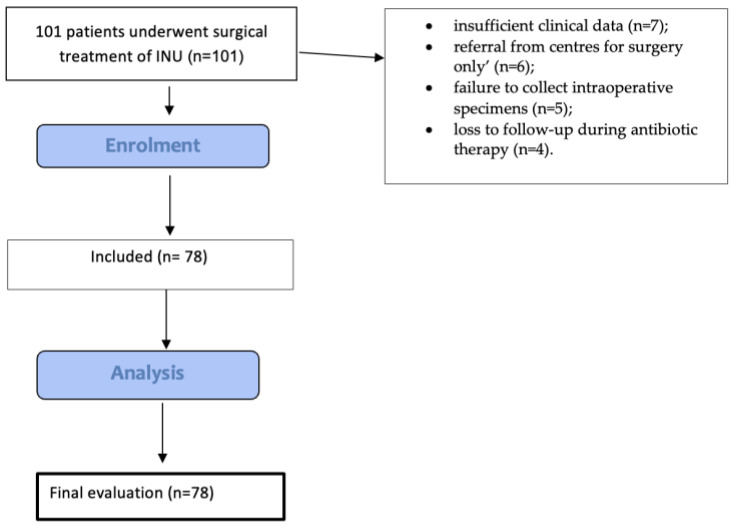
Flow diagram showing the included patients.

During the study period, 101 patients underwent surgical treatment of an INU; 23 were excluded because of the unavailability of sufficient clinical data (n = 7), referral from other centres for surgery only (n = 6), failure to collect intraoperative specimens (n = 5), and loss to follow-up during antibiotic therapy (n = 4) (Figure 1).

Overall, 78 patients were included; their characteristics are summarized in Table 1.

Briefly, 73% of the patients were male; their median age was 43 years; their median Charlson index was 0; 32% reported a history of an open fracture; and the non-union most frequently involved the leg. At the time of index surgery, 43.5% of patients had already undergone one or more treatment attempts at another centre after the diagnosis of INU.

**Figure 2:**
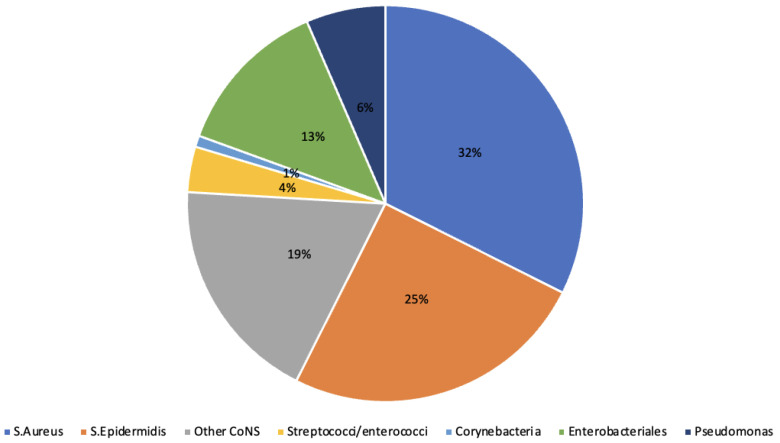
Pie chart: isolated microorganisms.

Index surgery consisted of debridement and hardware removal; external fixation was the most frequent approach to achieve bone stability; seven patients needed a bone graft. Intraoperative cultures were negative in 10 cases; polymicrobial infection was found in 23 cases (29%). The most common microorganisms were coagulase-negative staphylococci, followed by *Staphylococcus aureus* (Table 2, Figure 2).

Empirical antimicrobial therapy was started in the immediate post-operative phase in sixty-five cases, and it was not active against the isolated pathogen in two cases only (caused by K.pneumoniae and MRSE, respectively). In 13 patients (16%), antimicrobial therapy was started only after the results of intraoperative cultures and/or histopathology, because at the initial evaluation, the suspicion of an INU was low.

Teicoplanin was the most commonly prescribed drug in empirical therapy regimens (54 patients), and it was associated with piperacillin/tazobactam in 32 cases and with a fluroquinolone in 10 cases. After culture results, empirical antibiotic therapy was simplified to an oral regimen in 33 cases. Rifampicin was used in 49 out of 62 patients with *Staphylococcus* spp. cultured from intraoperative samples; the reasons for not using rifampicin were interaction with other drugs chronically used by the patient (n = 5), resistance pattern of the isolate (n = 4), and impaired liver function (n = 3). The median duration of antibiotic therapy was 54 days [IQR 41–63].

Overall, 25 patients underwent at least one more surgery, 10 underwent reconstructive surgery, and 15 underwent further debridement due to the persistence of non-union and/or local signs of infection. In the case of non-union, the infected area was resected and reconstructed through the Ilizarov technique.

At the 24-month follow-up from index surgery, sixteen patients had experienced clinical failure: thirteen (16.6%) presented with the persistence of local signs of infection (in two cases, infection recurred after reconstruction) and three (3.8%) had undergone amputation. Overall, clinical success was achieved in 62 patients (79%). The median duration of follow-up for patients who had achieved clinical success at 24 months from index surgery was 42 months (IQR 37–57); in this group, we observed no recurrence of infections.

Logistic regression analysis of risk factors for clinical failure identified BMI and MRSA infection as factors associated with clinical failure (*p* = 0.01 and *p* = 0.04, respectively) (Table 3).

## 3. Discussion

In this study, we described a cohort of patients with INUs managed at an ID referral centre and we found that, with a standardized management of antibiotic therapy, BMI and MRSA were the independent risk factors for clinical failure.

Fracture non-union is one the most challenging complications of trauma surgery; it is defined as the presence of a fracture that is non-united at nine months or has not healed within the expected time, or as a lack of progression of fracture healing on sequential radiographs [8]. Although infection is a well-known complication after fracture fixation, only recently did a consensus group publish and update a definition of fracture-related infections based on confirmatory and suggestive diagnostic criteria, which also applies to INUs [7]. While a definition has recently been provided, formal guidelines on the medical and surgical treatment of INUs are still lacking and, in clinical practice, management strategies are often extrapolated from those of other implant-related infections, in particular prosthetic joint infections (PJIs) [1,9]. However, there are major differences between PJIs, which usually follow an elective primary surgery, and INUs, which usually follow an emergency primary surgery and may occur in the presence of multiple fracture patterns and different degrees of soft tissue injury [7,10].

The absence of a clear definition of fracture-related infections has hampered the interpretation of research in this field, and it may be particularly useful to provide information on the epidemiology and outcome of INUs diagnosed following a standardized definition.

The main epidemiological findings of this study are consistent with previous data. Our population largely consists of young males without comorbidities; indeed, INUs often follow high-energy traumas resulting in open fractures (road accidents, workplace injuries, sport trauma), which are more common among young males [11,12].

A significant number of infections (29%) were polymicrobial; the most frequently involved microorganisms were staphylococci, Enterobacterales, and *Pseudomonas* spp. [13]. The proportion of culture-negative infections was 13%, similar to that reported in other studies using sonication [14,15].

With respect to antibiotic therapy, recent recommendations suggest using a lipo/glycopeptide plus an agent active against GNB as an empirical regimen, to be started immediately after debridement [16]. In our cohort, the high overall prevalence of methicillin resistance justified the usage of teicoplanin as an anti-Gram-positive agent in empirical therapy. Teicoplanin was preferred over vancomycin because of its tolerability and a more convenient schedule of administration [17]. Its combination with another drug active against GNB (levofloxacin or piperacillin/tazobactam) was justified by the isolation of Enterobacterales or *Pseudomonas* spp. in 19.5% of cases. A third of these isolates were resistant to quinolones, so we recommend caution in the usage of these molecules, which should be prescribed as empirical therapy only after an accurate evaluation of the risk factors for antibiotic resistance (i.e., comorbidities, previous hospitalizations, exposure to antibiotics, previous debridement, previous cultures).

The overall success rate in our cohort was 79%, which is in line with previous reports [18,19]. This is a satisfactory result, considering that near half of the patients were referred from other centres after the failure of at least one surgical approach. However, higher success rates have been reported when patients were managed by a dedicated multidisciplinary team [20,21,22]. Our patients were managed by an ID team skilled in the management of bone and joint infections, but surgery was performed by several orthopaedic teams; it is possible that the outcome would have been better with the creation of a dedicated orthopaedic team consisting of surgeons who are experts in the management of infected non-unions.

Among the patients’ baseline characteristics, only BMI was associated with clinical failure. A higher BMI may be associated with difficulties in stabilizing a fracture, and liponecrosis may result in more tissue inflammation and more significant post-surgical drainage. Moreover, obesity modifies the distribution of antibiotics to tissues, with a risk of under-dosing, especially when it is not possible to perform therapeutic drug monitoring [23]. Previous studies have reported that a higher BMI is a risk factor for fracture non-union and for infectious complications [11,24], but its impact on the outcome of INUs has not been extensively investigated so far.

MRSA infection was associated with a higher risk of clinical failure despite active empirical antimicrobial therapy with teicoplanin. MRSA is associated with a high risk of chronic and relapsing bone and joint infections due to its persistence mechanism (e.g., internalization in osteoblasts). As a consequence, not only the intrinsic antistaphylococcal activity, antibiofilm efficacy, and bone penetration of antibiotics but also their ability to eradicate the intracellular reservoir have to be taken into account when choosing antistaphylococcal therapy for bone and joint infections. These considerations suggest a potential role for other rapidly bactericidal anti-MRSA drugs (e.g., daptomycin) or combination therapy for the empirical therapy of INUs [25,26].

The position and type of the non-union and the time from the primary surgery were not associated with outcomes, suggesting that correct medical and surgical management may be effective even years after the fracture.

The main drawback of the present study is its retrospective nature, which may have introduced several biases, such as selection and recall biases. Additionally, our study has other limitations. First, the number of patients enrolled is limited, but it is comparable to those in previously published studies. Moreover, we provide information about epidemiology, microbiology, and overall clinical management, including antibiotic therapy, while previous studies were focused mainly on surgical treatment [27,28,29,30]. Second, this is a single-centre study, and our findings may be influenced by local epidemiology and surgical practices, with limited possibility to generalize the results. Third, we could not recover data about the grade of fracture exposition and the extension of bone and soft tissue loss, factors that may have an impact on clinical outcome. Finally, differences in surgical technique and expertise could have significantly influenced the outcomes.

To conclude, our findings highlight that a standardized medical and surgical management of INUs can lead to satisfactory results even years after the fracture and after the failure of previous treatment approaches.

Following a standardized management of antibiotic therapy by an expert ID consultant team, patient BMI and MRSA infection were associated with an increased risk of treatment failure, but further studies are needed to establish causality; ideally, a multicentre prospective study is necessary to validate these results across different populations and settings.

Further studies are warranted, especially ones that more extensively investigate the risk factors for unfavourable outcomes and that study the potential impact of surgical treatment performed by surgeons specifically skilled in the management of infected non-unions and accompanied by more aggressive antibiotic regimens for empirical therapy, such as daptomycin plus beta-lactams or daptomycin plus phosphomycin.

## 4. Materials and Methods

### 4.1. Study Design and Setting

We conducted a retrospective observational study including all consecutive adult patients with INUs treated at the Infectious Disease Unit of the IRCCS Azienda Ospedaliero-Universitaria di Bologna, Italy, from January 2012 to June 2018.

All patients aged ≥18 years with a confirmed INU (for a definition, see the Definition section) treated in the abovementioned period were eligible for this study; patients were excluded in case of the unavailability of complete clinical data, failure to collect intraoperative specimens, and follow-up of less than 12 months.

All patients underwent medical and surgical treatment at a referral centre for bone and joint infections, with strict collaboration between ID specialists, orthopaedic surgeons, and microbiologists. A dedicated ID consultant team performing more than 2500 bedside consultations and more than 600 ambulatory visits/year in this specific setting during the study period prescribed and monitored the antibiotic therapy. Surgical procedures were performed at three different orthopaedic centres in Bologna in collaboration with the abovementioned ID consultant team.

Surgery consisted of debridement, fixation device removal/exchange, and intraoperative sampling for histology and culture; the surgical technique was chosen by the surgical team according to type and site of the previous fracture, burden of bone loss, and quality of soft tissues. No antibiotic therapy was prescribed before surgery and any potential antibiotics were withheld for at least 14 days before surgery. Empirical broad-spectrum antibiotic therapy was started immediately after surgery, including coverage of methicillin-resistant *Staphylococcus* spp. and an agent active against Gram-negative bacilli (GNB) when an open fracture was reported in the patient history. Whenever possible, antibiotic therapy was de-escalated according to culture results and continued for 6 to 8 weeks after index surgery.

During antibiotic therapy, weekly assessment of full blood chemistry, including inflammatory markers, was recommended. After the discontinuation of antibiotics, a monthly assessment of C-reactive protein was carried out for 12 months and then repeated at 18 and 24 months after index surgery (first surgery performed in the abovementioned orthopaedic centres).

Outcomes were assessed at 24 months after index surgery.

### 4.2. Intraoperative Sampling and Processing of Study Specimens

Intraoperative specimens were collected during surgery in all cases; three to five bone samples were taken for culture and three for histopathology. These were harvested with dedicated sterile gouge pliers that were changed between each sampling.

Samples were processed at the Microbiology Laboratory of IRCCS Istituto Ortopedico Rizzoli and and IRCCS Azienda Ospedaliero-Universitaria di Bologna; they were handled in a class 2 biological safety cabinet to reduce the risk of contamination and the number of manipulations of the samples. Solid media and enrichment broth were both utilized for culturing samples, and the incubation time was at least five days for aerobic cultures and up to fourteen days for anaerobic ones. Removed implants were processed with sonication or dithiothreitol. In case of a positive culture, the microorganisms were identified using manual routine microbiology test procedures (Gram staining, catalase, coagulase, oxidase, indole) and an automated technique with a Vites-2 system (bioMérieux, Marcy l’Etoile, Craponne, France). Antimicrobial susceptibility was assessed according to the EUCAST (European Committee on Antimicrobial Susceptibility Testing) recommendations; minimum inhibitory concentration (MIC) was provided for each tested antimicrobial.

Samples for pathology were fixed in buffered 4% formaldehyde solution, decalcified with dilute hydrochloric acid and chelating agents, and then embedded in paraffin. Tissue blocks were then cut and stained with hematoxylin–eosin. The pathologist who examined the samples was unaware of the culture results.

### 4.3. Definitions

INU was defined as a fracture non-union in the presence of at least one of the following clinical, microbiological, and histopathological criteria consistent with infection: presence of a sinus tract; isolation of phenotypically indistinguishable microorganisms from the culture of at least 2 separate deep tissue/implant specimens; presence of >5 PMNs/HPF at histopathology of intraoperative specimens [6].

Empirical antimicrobial therapy was considered active if intraoperative cultures yielded microorganisms that were susceptible to the prescribed drugs.

We defined “clinical failure” as a patient’s death, need for amputation, or one of the following:

(a) In patients who did not need reconstructive surgery, persistence of local signs of infection (sinus tract, rubor, tumor, dolor, calor) and/or persistence of the non-union at 24-month follow-up;
(b) In patients who underwent reconstructive surgery, presence of local signs of infection (sinus tract, rubor, tumor, dolor, calor) or need for further surgical debridement or suppressive antibiotic therapy after reconstruction.

### 4.4. Data Collection

We retrospectively collected data by revising medical records. We acquired data on demographics and comorbidities; presence of potential risk factors for non-healing of the fracture (cigarette smoking, body mass index, use of corticosteroids, intravenous drug usage); characteristics of the fracture and initial management (open fracture, type of synthesis, use of bone graft, number and reasons of subsequent surgeries after primary synthesis); index surgery (technique, application of bone graft, Gram stain and culture results of intraoperative samples); antimicrobial therapy (drug, dosage, route of administration, duration); and outcome.

### 4.5. Statistical Analysis

Descriptive statics were obtained for all variables considered; categorical variables were expressed as absolute numbers and proportions, continuous variables as mean and standard deviation if normally distributed and as median and interquartile range (IQR) if non-normally distributed.

Patients who achieved clinical success were compared with those who experienced clinical failure: the chi-square test or Fisher exact test was used to compare categorical variables and the Mann–Whitney *U*-test for continuous variables, as appropriate.

To assess risk factors for clinical failure, we performed a univariate and multivariate logistic regression analysis; variables with a *p* value < 0.1 at univariate analysis were included in the multivariate model. A *p*-value of <0.05 was considered statistically significant.

Multicollinearity was assessed through the analysis of the variance inflation factor.

Statistical analyses were performed with SPSS, version 23 (SPSS, Chicago, IL, USA).

## Figures and Tables

**Table 1:** Demographics and clinical characteristics of the study population.

	All Patients (n = 78)	Clinical Success (n = 62)	Clinical Failure (n = 16)	*p*
Baseline characteristics				
Age (years)—median (IQR)	43 (34–56)	41 (32–55)	54 (40–63)	0.04
Males—n° (%)	57 (73.1%)	45 (73%)	12 (75%)	0.84
BMI (Kg/m^2^)—median (IQR)	28 (24–30)	28 (24–29)	30 (26–36)	0.05
Cigarette smokers—n° (%)	29 (37.2%)	22 (35.5%)	7 (44%)	0.54
IVDU—n° (%)	4 (5.1%)	2 (3%)	2 (12.5%)	0.18
CCI—median (IQR)	0 (0–2)	0 (0–1)	1 (0–3)	0.12
Diabetes—n° (%)	10 (13)	7 (11)	3 (19)	0.43
Chronic steroid use—n° (%)	4 (5.1%)	4 (7.3%)	0 (0%)	0.18
Characteristics of the fracture and surgery before referral				
Open fracture—n° (%)	32 (41)	25 (40)	97(44)	0.80
Site of fracture—n° (%)				0.90
Shin bones *	50 (64)	39 (63)	11 (69)	
Femur	12 (15)	10 (16)	2 (12.5)	
Arm §	16 (20.5)	13 (21)	3 (19)	
Primary fixation—n° (%)				0.21
Plate and screw	31 (40)	24 (44)	7 (39)	
Intramedullary nail	20 (26)	19 (31)	1 (6)	
External fixation	19 (24)	14 (22)	5 (31)	
Others	8 (10)	5 (8)	3 (19)	
Index surgery				
Time from primary fixation to index surgery, months—median (IQR)	13 (6–27)	11 (6–22)	20 (11–40)	0.07
Type of surgery				
Debridement + cast	18 (23)	12 (19)	6 (37.5)	0.12
External fixation	45 (58)	36 (58)	9 (56)	0.89
Internal fixation	15 (19)	14 (22)	1 (6)	0.17
Use of bone graft—n° (%)	7 (9%)	7 (11%)	0 (0%)	0.15
Etiology and antibiotic therapy				
MRSA	11 (14)	6 (10)	5 (31)	0.04
Gram-negative	12 (15)	9 (14.5)	3 (19)	0.70
Polymicrobial infection—n° (%)	21 (27)	16 (26)	5 (31)	0.66
Culture-negative—n° (%)	10 (13)	8 (13)	2 (12.5)	1
Antibiotic therapy—n° (%)				
IV only	32 (41)	23 (37)	9 (56)	0.16
Oral step-down	33 (42)	26 (42)	7 (44)	0.89
Oral only	13 (17)	13 (21)	0 (0)	0.06
Duration of AT, days—median (IQR)	54 (41–63)	52 (40–63)	56 (50–64)	0.19

IQR: interquartile range, BMI: body mass index, IVDU: intravenous drug user, CCI: Charlson Comorbidity Index, MRSA: methicillin-resistant *Staphylococcus aureus*, IV: intravenous, AT: antibiotic therapy. * Tibia n = 45, tibia + fibula n = 5; § Humerus n = 11, radius + ulna n = 3, radius = 1, ulna = 1.

**Table 2:** Microorganisms isolated during index surgery.

	All Isolates (n = 108)
Gram-positive	
*S. aureus*	35 (32%)
MR	11
MS	24
*S. epidermidis*	27 (25%)
MR	21
MS	6
Other CoNS	20 (18.5%)
MR	12
MS	8
Streptococci/enterococci	4 (3%)
Corynebacteria	1 (1%)
Gram-negative	
Enterobacteriales	14 (13%)
FQ-R	6
FQ-S	8
*Pseudomonas*	7 (6.5%)
FQ-R	2
FQ-S	5

MR: methicillin-resistant, MS: methicillin-sensible, CoNS: coagulase negative staphylococci, FQ-R: resistant to fluoroquinolones, FQ-S: sensible to fluoroquinolones.

**Table 3:** Univariate and multivariate logistic regression analysis of factors associated with clinical failure at 24 months’ follow-up.

	OR (95% CI)	*p*	aOR (95% CI)	*p*
Age	1.03 (0.99–1.07)	0.07	1.03 (0.99–1.07)	0.13
Male sex	0.88 (0.25–3.11)	0.84		
BMI	1.11 (1.01–1.22)	0.03	1.15 (1.03–1.28)	0.01
CCI	1.30 (0.94–1.79)	0.10		
Diabetes	1.81 (0.41–7.98)	0.43		
Smoking	1.41 (0.46–4.32)	0.54		
Open fracture	1.15 (0.38–3.49)	0.80		
Site of fracture				
Leg	1.29 (0.40–4.20)	0.66		
Femur	0.74 (0.14–3.78)	0.72		
Arm	0.87 (0.21–0.35)	0.84		
Time from primary fixation to IS	1.01 (1–1.02)	0.08	1.01 (0.99–1.02)	0.33
Surgery				
Debridement + cast	2.5 (0.75–8.23)	0.13		
External fixation	0.92 (0.20–2.81)	0.89		
Internal fixation	0.23 (0.02–1.88)	0.17		
Polymicrobial infection	1.30 (0.39–4.34)	0.66		
Gram-negative	1.35 (0.32–5.74)	0.67		
MRSA infection	4.24 (1.09–16.38)	0.04	5.35 (1.06–26.92)	0.04
Culture negative	0.96 (0.18–5.05)	0.96		
Step-down oral antibiotic therapy	1.07 (0.35–3.26)	0.89		

BMI: body mass index, CCI: Charlson Comorbidity Index, IS: index surgery, MRSA: methicillin-resistant *Staphylococcus aureus*.

## Data Availability

The raw data supporting the conclusions of this article will be made available by the authors on request.
